# A Novel Oral Film Formulation for Jujuboside: Response Surface Optimization of Preparation Parameters and Performance Evaluation

**DOI:** 10.3390/foods15142413

**Published:** 2026-07-08

**Authors:** Yu Chen, Shujing Xuan, Beizhi Zhang, Fuzhi Xie, Nannan Chen, Qing Zhang, Bei Fan, Fengzhong Wang, Liang Zhang

**Affiliations:** 1 National Center of Technology Innovation for Comprehensive Utilization of Saline-Alkali Land, Dongying 257000, China; cynthia1070@163.com; 2 Institute of Food Science and Technology, Chinese Academy of Agricultural Sciences, Beijing 100193, China; m14753632786@163.com (S.X.); zhangbeizhi777@163.com (B.Z.); xiefuzhi2002@163.com (F.X.); chennan0114@gmail.com (N.C.); 13345033761@163.com (Q.Z.); fanbei517@163.com (B.F.); 3 College of Food Science and Technology, Huazhong Agricultural University, Wuhan 430070, China; 4 College of Food Science and Engineering, Qingdao Agricultural University, Qingdao 266000, China; 5 Institute of Food and Nutrition Development, Ministry of Agricultura and Rural Affairs, Beijing 100081, China

**Keywords:** jujuboside, oral film, response surface method, disintegration time, content uniformity

## Abstract

Jujuboside is an active saponin with anxiolytic and sedative effects, but its oral bioavailability is extremely low. This study prepared an oral film to achieve rapid absorption through the oral mucosa. Using jujuboside as the raw material, the mass ratio of vinyl alcohol polymer to microbial polysaccharide, the ratio of propylene glycol to glycerol and the amount of cellulose ether were selected as influencing factors. On the basis of single-factor experiments, the response surface method was employed to optimize the preparation process of jujuboside oral films, and the effects of these factors on disintegration time and flexibility of the films were investigated. Meanwhile, the content uniformity of jujuboside A and γ-aminobutyric acid in the films was determined by high-performance liquid chromatography. Results showed that the optimized jujuboside oral film had complete molding, a smooth surface, good flexibility, a disintegration time of 25 s in simulated oral environment, and acceptable content uniformity of jujuboside A and γ-aminobutyric acid. The oral film can disintegrate rapidly and release the drug in the oral cavity, and is designed for oromucosal delivery. This oral film is convenient to take, particularly suitable for children, the elderly and patients with dysphagia, providing a new strategy for the clinical application of jujuboside.

## 1. Introduction

Insomnia, a common yet often neglected disorder, significantly impairs both physical and mental health. Studies have shown that approximately 10–30% of the general population suffers from insomnia [1]. Although pharmacotherapy is the standard of care, its long-term use often causes adverse effects such as dependency, tolerance, and cognitive deficits, driving an urgent need for safer natural alternatives [2,3,4]. Zizyphi Spinosae Semen (ZSS) is a popular traditional Chinese herbal food for treating fright palpitations, insomnia, and dreaminess [5]. ZSS has also been recognized as one of the first medicinal botanical drugs included in China’s official list of substances with dual functions of medicine and food [6]. In traditional Chinese dietary therapy, ZSS is commonly incorporated into daily meals to promote health and well-being [7]. For example, ZSS is often cooked with rice, lily bulb, or longan pulp to prepare a soothing congee that alleviates restlessness and improves sleep quality. Additionally, it can be stewed with pork heart or lean meat to produce a nourishing soup, or stir-fried, ground into powder, and incorporated into baked goods such as biscuits and cakes, as well as honey-based beverages for a calming effect [8]. Aside from these direct culinary uses, ZSS has also been explored as a functional ingredient in modern food products: supplementation of yogurt with water-soluble ZSS extract (SZSE) has been shown to reduce syneresis and promote the formation of a more compact and uniform casein gel [9]. Together, these traditional and contemporary applications highlight the unique value of ZSS as a substance with medicine-food homology.

The major bioactive components of ZSS, jujuboside A and jujuboside B, are triterpene saponins that exhibit sedative, hypnotic, and anti-anxiety activities [10]. However, the natural content of jujubosides in ZSS is relatively low, which limits their application in biomedicine and health foods [11]. To isolate jujubosides from ZSS, several extraction methods have been developed. Conventional approaches include reflux extraction using ethanol, followed by concentration and purification with water-saturated n-butanol or macroporous adsorption resin column chromatography, and finally freeze-drying to obtain the saponin extract [12]. Advanced techniques, such as ultrasonic-assisted alcohol extraction, have been reported to significantly increase jujuboside yield by up to 482.14% [13]. Nevertheless, jujubosides are inherently sensitive to heat, oxygen, and various food processing conditions, which restricts their direct application in functional food products [14]. Therefore, encapsulation technology represents a promising strategy to improve the stability, controlled release, and bioavailability of ZSS saponins in the contexts of food preservation and functional food development.

Jujuboside A is one of the key saponins in Zizyphi Spinosae Semen (ZSS) and is the main component responsible for its anti-anxiety and sedative effects [15]. Recent research has revealed that by targeting GABA receptors and activating PVT-region PV/SST neuronal activity, jujuboside A ameliorates insomnia and restores physiological sleep–wake rhythms [16]. In addition, jujuboside A ameliorates insomnia by restoring mitochondrial oxidative phosphorylation, regulating the mitochondrial permeability transition pore (mPTP) to maintain mitochondrial homeostasis, and alleviating mitochondrial structural damage [17]. However, in vivo studies have demonstrated that gastrointestinal absorption results in low bioavailability of jujuboside A. Jujuboside A is hydrolyzed mainly in the stomach and subsequently absorbed across distinct intestinal segments [5]. The drawback prompted us to explore whether other new strategies could serve as viable alternatives for the clinical application of jujuboside.

Compared with other mucosal routes, the oromucosal route exhibits higher permeability, attributable to the thinner and less keratinized oral epithelium. Moreover, features such as enhanced vascularization, negligible oral enzymatic inactivation, and the bypassing of hepatic first-pass metabolism all contribute to achieving more rapid drug absorption and a faster onset of action following oromucosal delivery [18]. Unlike liquid formulations (e.g., gargles and sprays), oral films exhibit improved adhesion and resistance to salivary washout [19,20]. In contrast to local injections, oral films are noninvasive and user-friendly [21]. Furthermore, while semi-solid formulations extend drug action to some extent, they remain susceptible to saliva erosion, whereas oral films provide more sustained drug retention and release [22]. Consequently, numerous novel multifunctional oral films have emerged in recent years. However, despite the surge in research on multifunctional oral films, their clinical translation remains limited due to challenges including intricate design, laborious fabrication, and unresolved biosafety issues [22]. Based on the characteristics of oral films, we hypothesize that different ratios of matrix solutions and preparation methods can enable more efficient fabrication of oral films. Furthermore, on the basis of this efficient fabrication, the disintegration time of the oral films can be shortened while achieving good flexibility.

To evaluate the viability of oral films for jujuboside, the jujuboside oral films were prepared using different matrix ratios. The prepared films underwent a series of single-factor and response surface experiments to determine their optimal parameters. Their disintegration time, flexibility, and content uniformity were subsequently evaluated.

## 2. Materials and Methods

### 2.1. Materials

In this work, we developed a jujuboside–GABA combinatory film (a Zizyphi Spinosae Semen (ZSS)-inspired functional formulation) rather than a single-component jujuboside film. Jujuboside (4%) was purchased from Shaanxi Yuzhou Biotechnology Co., Ltd. (Xi’an, Shaanxi, China). Xylitol was purchased from Shandong Futian Pharmaceutical Co., Ltd. (Dezhou, Shandong, China). Mannitol (D-mannitol) was purchased from Qingdao Mingyue Algae Group Co., Ltd. (Qingdao, Shandong, China). γ-aminobutyric acid from Haozhou Boguang Biotechnology Co., Ltd. (Haozhou, Anhui, China). Titanium dioxide (purity 99.5%) was purchased from Jiangsu Ruikanglai Technology Co., Ltd. (Nanjing, Jiangsu, China). Propylene glycol was purchased from Lianyungang Xin’ai Food Technology Co., Ltd. (Lianyungang, Jiangsu, China). Edible glycerin was purchased from Guangzhou Kangben Biotechnology Co., Ltd. (Guangzhou, China). Cellulose ethers, vinyl alcohol polymers and microbial polysaccharides were purchased from Beijing Guangda Hengyi Technology Co., Ltd. (Beijing, China). All of the above are food-grade food additives. PBS phosphate buffer (0.01 mol/L, pH 7.0, sterile) was purchased from Shanghai Yuanye Biotechnology Co., Ltd. (Shanghai, China). Formic acid, methanol, acetonitrile (chromatographic grade, Fisher, Shanghai, China).

### 2.2. Sample Development

#### 2.2.1. Preparation of the Solution

Active ingredient extract solution: 1 g of γ-aminobutyric acid and 1 g of jujuboside were weighed successively and dissolved in 5 mL of water at room temperature. Ultrasound was applied to ensure complete dissolution and uniform mixing of the extract.

Matrix solution: Vinyl alcohol polymers were added to 50 mL of water at room temperature; the mixture was stirred by a magnetic stirrer (at 65 °C) for approximately 30 min to allow swelling and complete dissolution. While maintaining a water bath at 65 °C, different amounts of cellulose ether, microbial polysaccharides, propylene glycol, glycerol, 50 mg of mannitol, 50 mg of xylitol and 5 mg of titanium dioxide were added sequentially. The mixture was stirred evenly to form a stable solution.

The active ingredient extract solution was mixed with the matrix solution, and ultrasound was applied to assist in degassing.

#### 2.2.2. Film Forming

**Figure 1:**
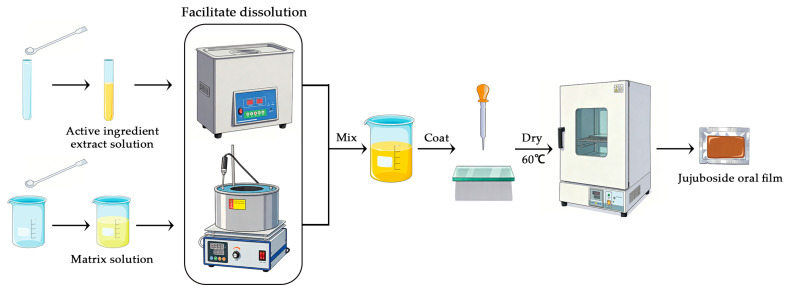
Illustration of the preparation process of Jujuboside Oral Film.

The mixed solution was uniformly coated onto a flat glass plate or non-stick paper, the thickness before drying is approximately 3–4 mm. After coating, the film needed to be hot air dried at 60 °C for 180 min until the film surface became dry and tough. The dried film had a final thickness of 0.5 ± 0.25 mm and a dry mass of approximately 120 mg per cm^2^ (calculated from total dry weight and coating area). The dried film was cut into pieces according to the required specifications. A common size was suitable for oral use (e.g., 1 cm × 1 cm). The cut strips showed good mass uniformity: average weight 120 ± 5 mg (RSD < 5%) and drug loading of 5.0 mg per strip as determined by HPLC. Additional characterization gave surface pH 6.8 ± 0.2, moisture content < 3.0%, and water activity 0.45 ± 0.05. After cutting, the pieces were packaged and stored in an environment with low humidity to prevent the film from absorbing moisture and becoming soft. Aluminum foil bags were selected as the packaging material (Figure 1). Stability tests under 25 °C/60% RH for 6 months showed that the film remained stable, with drug content > 98% of initial and no significant changes in appearance or dissolution profile.

#### 2.2.3. Sample Pretreatment for Determination of γ-Aminobutyric Acid in Oral Films

Biological samples stored at 4 °C were taken out, weighed, and fully ground into powder using a low-temperature grinder (50 Hz, 1 min). To the ground sample, 1.2 mL of 0.1% formic acid aqueous solution (*v*/*v*) was added for extraction, followed by vortex mixing and ultrasonication for 30 min. The resulting mixture was centrifuged at 12,000 r/min for 5 min. An aliquot of 20 μL of the supernatant was transferred to a new centrifuge tube and mixed with 80 μL of acetonitrile. The mixture was filtered through a 0.22 μm organic membrane, placed into an autosampler vial, and then analyzed on an UPLC-MS/MS system.

#### 2.2.4. Sample Pretreatment for Determination of Jujuboside A in Oral Films

Biological samples stored at 4 °C were taken out, weighed, and fully ground into powder using a low-temperature grinder (50 Hz, 1 min). To the ground sample, 1.2 mL of pure methanol was added for extraction, followed by vortex mixing and ultrasonication for 30 min. The resulting mixture was centrifuged at 12,000 r/min for 5 min. An aliquot of 20 μL of the supernatant was transferred to a new centrifuge tube and mixed with 80 μL of acetonitrile. The mixture was filtered through a 0.22 μm organic membrane, placed into an autosampler vial, and then analyzed on an UPLC-MS/MS system.

#### 2.2.5. Instrument Conditions

##### Chromatographic Conditions for γ-Aminobutyric Acid

The analysis was performed on an ExionLC AC chromatograph equipped with a HYPERSIL GOLD C18 column (3 μm, 2.1 mm × 100 mm) (Framingham, MA, USA). The injection volume was 1.0 μL, and the column temperature was maintained at 40 °C. The mobile phase consisted of A (0.1% formic acid) and B (acetonitrile). The gradient elution program was as follows: 0–0.3 min, 96% A; 0.3–2 min, 96% to 70% A; 2–6 min, 70% to 0% A; 6–6.1 min, 0% to 96% A; and 6.1–10 min, 96% A. The flow rate was 0.2 mL/min, and the total run time was 10 min.

##### Chromatographic Conditions for Jujuboside A

The same chromatograph and column were used. The injection volume was 1.0 μL, and the column temperature was 40 °C. The mobile phase consisted of A (10 mM ammonium formate in water containing 0.1% formic acid) and B (acetonitrile). The gradient elution program was as follows: 0–2 min, 75% A; 2–6 min, 75% to 10% A; 6–8 min, 10% A; 8–8.1 min, 10% to 75% A; and 8.1–10 min, 75% A. The flow rate was 0.3 mL/min, and the total run time was 10 min.

##### Mass Spectrometry Conditions

Mass spectrometry was performed on a TRIPLE QUAD 4500 (Framingham, MA, USA) mass spectrometer equipped with an electrospray ionization (ESI) source. The collision gas (CAD) was set at 9 psi, curtain gas (CUR) at 30 psi, ion source gas 1 (GS1) at 50 psi, and ion source gas 2 (GS2) at 55 psi. The ion spray voltage was +5500 V in positive ion mode and −4500 V in negative ion mode. The ion source temperature was 550 °C. The scan mode was multiple reaction monitoring (MRM).

##### Method Validation

The method was validated for specificity, linearity, sensitivity, precision, accuracy, recovery, matrix effect, carryover, and stability. Specificity was confirmed by analyzing six batches of blank oral film matrices; no endogenous peaks interfering with the analytes were observed at the respective retention times.

Calibration curves were constructed in blank matrix over seven concentration levels. Good linearity was achieved for GABA in the range of 1–500 ng/mL and for jujuboside A in the range of 0.5–200 ng/mL, with coefficients of determination (R^2^) > 0.995 for all curves. The limits of detection (LOD, S/N = 3) were 0.3 ng/mL and 0.15 ng/mL, and the limits of quantification (LOQ, S/N = 10) were 1.0 ng/mL and 0.5 ng/mL for GABA and jujuboside A, respectively.

Intra-day and inter-day precision and accuracy were determined at LLOQ, low, medium, and high QC levels (*n* = 5). The relative standard deviations (RSD) were <8.3% (intra-day) and <9.8% (inter-day) for GABA, and <7.2% and <8.5% for jujuboside A. The relative errors (RE) ranged from −3.0% to 4.0% for GABA and from −2.0% to 3.5% for jujuboside A, all within the acceptance criteria of RSD < 15% and RE within ±15% (±20% at LLOQ).

Extraction recoveries and matrix effects were evaluated at three QC levels (n = 6). Mean recoveries were 82.6–91.5% for GABA and 78.3–88.4% for jujuboside A. The matrix effects, calculated as the ratio of peak areas in post-extraction spiked matrix to those in neat solutions, ranged from 89.7% to 106.8% for both analytes, indicating negligible ion suppression or enhancement. Carryover was examined by injecting blank acetonitrile immediately after the highest calibration standard; no significant residual peaks (<20% of LLOQ area) were detected.

Stability studies included short-term (24 h at room temperature), long-term (30 days at −20 °C), three freeze–thaw cycles, and autosampler storage (12 h at 4 °C) at LQC and HQC levels. The deviations from nominal concentrations were within ±15% for both analytes under all conditions, confirming their stability throughout the entire analytical workflow.

### 2.3. Analytics

#### 2.3.1. Grading Standard for Flexibility of Jujuboside Oral Film

Based on the flexibility performance of the Jujuboside oral film, the rating standards were established and are shown in Table 1. All scoring was performed by three trained evaluators under standardised conditions (25 °C, 50% RH), and the median score of the three readings was used as the final response.

#### 2.3.2. Measurement of Disintegration Time

Phosphate-buffered saline (PBS, 0.01 mol/L, pH 7.0, sterile) was selected as the medium because it mimics human saliva in ionic composition and is pharmacopeially recommended. A volume of 15 mL was used to ensure full immersion and uniform depth (pre-validated). The medium was preheated to 37 ± 1 °C in a 60-mm petri dish placed on an orbital shaker at 50 rpm. Each oral film was cut to 1 cm × 1 cm. The film was immersed, and timing started immediately. The disintegration endpoint was defined as the moment when no visible solid particles or film remnants remained against a white background. Each group was tested in triplicate (n = 3).

#### 2.3.3. Screening of Matrix Solution Conditions

A single-factor experimental design was adopted. The ratio of propylene glycol to glycerol was fixed at 1:1 (200 mg:200 mg), and the amount of cellulose ether was fixed at 125 mg. The effect of the mass ratio of vinyl alcohol polymers to microbial polysaccharides (1:1 = 312.5 mg:312.5 mg, 3:2 = 375 mg:250 mg, 3.5:1.5 = 437.5 mg:187.5 mg, 4:1 = 500 mg:125 mg, 4.5:0.5 = 562.5 mg:62.5 mg) on the Jujuboside oral film was investigated. The mass ratio of vinyl alcohol polymers to microbial polysaccharides was fixed at 3.5:1.5 (437.5 mg:187.5 mg), and the amount of cellulose ether was fixed at 125 mg. The effect of the ratio of propylene glycol to glycerol (0:4 = 0 mg:400 mg, 1:3 = 100 mg:300 mg, 1:1 = 200 mg:200 mg, 3:1 = 300 mg:100 mg, 4:0 = 400 mg:0 mg) on the Jujuboside oral film was investigated. The mass ratio of vinyl alcohol polymers to microbial polysaccharides was fixed at 3.5:1.5 (437.5 mg:187.5 mg), and the ratio of propylene glycol to glycerol was fixed at 1:1 (200 mg:200 mg). The effect of the amount of cellulose ether (25 mg, 75 mg, 125 mg, 175 mg, 225 mg) on the Jujuboside oral film was investigated. Three replicates were performed for each treatment group.

#### 2.3.4. Response Surface Design

On the basis of single-factor experiments, the response surface method was employed to optimize the preparation of Jujuboside oral films. The mass ratio of vinyl alcohol polymers to microbial polysaccharides (A), the ratio of propylene glycol to glycerol (B), and the amount of cellulose ether (C) were chosen as independent variables, while disintegration time and flexibility were taken as response variables. A three-factor, three-level central composite design (CCD) was adopted. The CCD consisted of 8 factorial points, 6 axial points (star points), and 6 center points, totaling 20 experimental runs. The axial distance alpha was set at 1.6818 to maintain rotatability. The coded and actual levels of the factors are presented in Table 2.

#### 2.3.5. Qualitative and Quantitative Methods

Qualitative analysis: The target compounds were identified using a triple quadrupole mass spectrometer in MRM mode by optimizing the standards to obtain characteristic ion pairs. Combined with the retention time and ion pair abundance ratio of the standards under the same liquid chromatography conditions, the target compounds could be precisely identified, effectively eliminating false-positive interference from complex matrices.

Quantitative analysis: A standard curve was constructed by plotting the peak areas of standard solutions at different concentration gradients, and the linear equation was obtained. The peak area of each chromatographic peak in the samples reflected the relative content of the corresponding substance. The quantitative analysis results of the target substance in all samples were eventually obtained by substituting the peak areas into the linear equation and the calculation formula. The formula was as follows:

Content of target substance in sample (μg/g) = (c × V × d)/m

where:

c = concentration of the sample obtained by substituting the integrated peak area into the standard curve (μg/mL);

V = volume of the extraction solution (mL);

m = sample weight (g);

d = dilution factor (if the sample solution is not further diluted, d = 1; if diluted, d represents the multiplier of the dilution, e.g., if 1 mL of extract is diluted to 10 mL, then d = 10).

Unit consistency note: To maintain the final result in μg/g (as reported in Table 6), the extraction volume V must be expressed in mL. If V is measured in μL instead, a conversion factor of 1000 must be included in the denominator to ensure unit consistency, as follows:

Content (μg/g) = (c × V × d)/(1000 × m)

where V is in μL.

### 2.4. Statistics

All experiments were performed with three technical replicates. Data was checked by the Shapiro–Wilk test and homogeneity of variances by Levene’s test. One-way ANOVA was performed using the GLM procedure of SAS 9.2. Means of different treatment groups were compared using Duncan’s multiple range test, with a significance level set at *p* < 0.05. Graphs were plotted with GraphPad Prism 8, and response surface analysis was carried out with Design Expert 10.0.7.

## 3. Results

### 3.1. Effects of Different Matrix Solution Conditions on Jujuboside Oral Films

**Figure 2:**
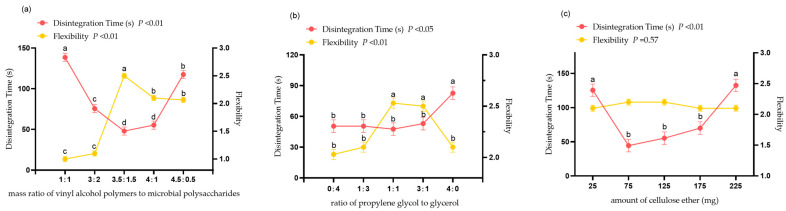
Effects of (**a**) the mass ratio of vinyl alcohol polymers to microbial polysaccharides, (**b**) ratio of propylene glycol to glycerol, and (**c**) amount of cellulose ether on Jujuboside Oral Films. Bars annotated with the same letters indicate no statistically significant difference between those groups; bars with entirely different letters indicate a significant difference (*p* < 0.05).

The effects of the mass ratio of vinyl alcohol polymer to microbial polysaccharide, the ratio of propylene glycol to glycerol, and the addition amount of cellulose ether on the disintegration time and flexibility of jujuboside oral films are shown in Figure 2. As illustrated in Figure 2, the shortest disintegration time of the oral films was observed at the mass ratio of vinyl alcohol polymers to microbial polysaccharides of 3.5:1.5 and 4:1 (Figure 2a), the ratio of propylene glycol to glycerol of 0:4, 1:3, 1:1 and 3:1 (Figure 2b), and the amount of cellulose ether of 75, 125 and 175 mg (Figure 2c). Under these conditions, the flexibility of the oral films was also closest to 3 (Figure 2a,b). Furthermore, the amount of cellulose ether added had no significant effect on the flexibility of the oral films, which had no significant differences among treatment groups (Figure 2c). Therefore, the treatment groups with the shortest disintegration time and optimal flexibility were selected for the subsequent response surface experiment.

The selected groups were: the mass ratio of vinyl alcohol polymers to microbial polysaccharides of 3:2, 3.5:1.5 and 4:1; the ratio of propylene glycol to glycerol of 0:4, 1:3 and 1:1; the amount of cellulose ether of 75, 125 and 175 mg.

### 3.2. Results of the Response Surface Experiment

**Figure 3:**
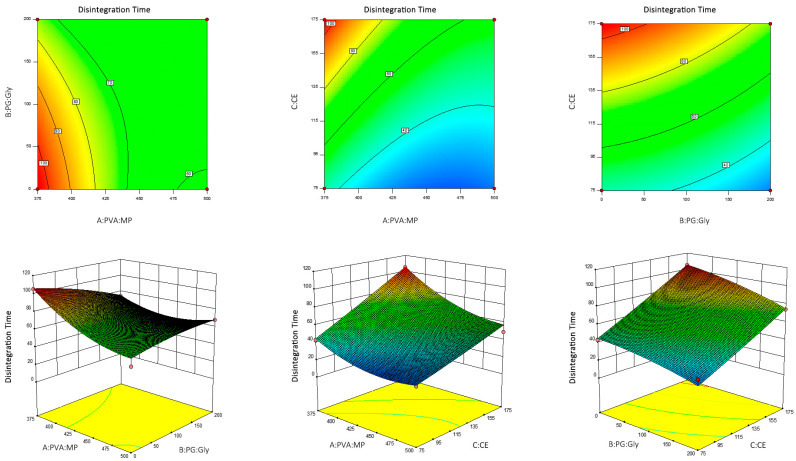
Response Surface Plots and Contour Plots of the Disintegration Time of Jujuboside Oral Films as a Function of the Mass Ratio of Vinyl Alcohol Polymer to Microbial Polysaccharides, the Ratio of Propylene Glycol to Glycerol, and the Amount of Cellulose Ether.

**Figure 4:**
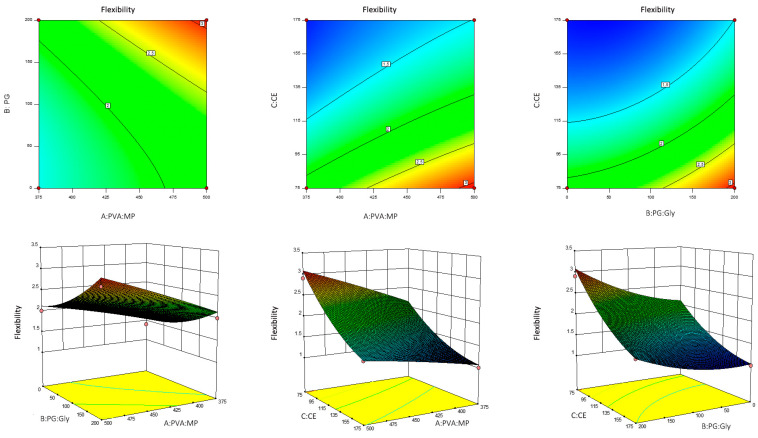
Response Surface Plots and Contour Plots of the Flexibility of Jujuboside Oral Films as a Function of the Mass Ratio of Vinyl Alcohol Polymer to Microbial Polysaccharides, the Ratio of Propylene Glycol to Glycerol, and the Amount of Cellulose Ether.

Based on the single-factor experimental results, the mass ratio of vinyl alcohol polymer to microbial polysaccharide (A), the ratio of propylene glycol to glycerol (B), and the amount of cellulose ether (C) were selected as independent variables, while the disintegration time (Y_1_)and flexibility (Y_2_) of Jujuboside oral films were taken as response values. A three-factor, three-level experimental design was carried out using the central composite design principle. The results are shown in Table 3. The response surface plots and contour plots for the disintegration time of Jujuboside oral films versus the factors are shown in Figure 3, and those for flexibility are shown in Figure 4.

The ANOVA results for the two response variables are summarized in Table 4 and Table 5. For disintegration time (Y_1_), the linear coefficients of A (*p* = 0.0011) and C (*p* < 0.0001) exerted significant main effects, while the linear term B (*p* = 0.1946) showed no significant influence. Regarding the interaction effects, the cross-terms AB (*p* = 0.0003) and AC (*p* = 0.0040) were significant, indicating that the effect of factor A on disintegration time was modulated by both B and C, whereas the BC interaction was not significant (*p* = 0.1653). For the quadratic effects, only A^2^ (*p* < 0.0001) reached significance; B^2^ (*p* = 0.0544, borderline) and C^2^ (*p* = 0.6014) were not statistically significant at the 0.05 level. For flexibility (Y_2_), all three linear coefficients A (*p* = 0.0008), B (*p* = 0.0005), and C (*p* < 0.0001) showed significant main effects. In contrast, none of the interaction terms (AB, AC, or BC) were significant (*p* > 0.05 for all), suggesting that the effects of the three formulation factors on flexibility were largely independent of each other. Among the quadratic terms, only B^2^ (*p* = 0.0068) and C^2^ (*p* = 0.0021) exhibited significant curvature effects, whereas A^2^ (*p* = 0.6332) did not show a significant quadratic contribution.

Table 4 is the ANOVA table for the disintegration time of Jujuboside oral films. From Table 4, the model *p* < 0.0001 indicates high significance, showing that the equation obtained from the model fits the actual data very well, and the model can be used to design experiments for optimizing the preparation process of the Jujuboside oral films. The lack of fit *p* = 0.0766 > 0.05 is not significant, indicating that the experimental error is small and the model residuals arise from random error. The coefficient of determination R^2^ = 0.9698, and the difference between R^2^Adj = 0.9426 and R^2^Pred = 0.8018 is less than 0.2, indicating a high correlation between experimental values and predicted values, and the response surface design is reasonable. According to the F value and *p*-value, the order of influence on the disintegration time is: the amount of cellulose ether (C) > the mass ratio of vinyl alcohol polymer to microbial polysaccharides (A) > the ratio of propylene glycol to glycerol (B). The interactions between A and B, and between A and C were significant (*p* < 0.05).

Table 5 is the ANOVA table for the flexibility of Jujuboside oral films. From Table 5, the model *p* < 0.0001 indicates high significance, showing that the equation obtained from the model fits the actual data very well, and the model can be used to design experiments for optimizing the preparation process of the Jujuboside oral films. The lack of fit *p* = 0.2065 > 0.05 is not significant, indicating that the experimental error is small and the model residuals arise from random error. The coefficient of determination R^2^ = 0.9509, and the difference between R^2^Adj = 0.9067 and R^2^Pred = 0.7220 is less than 0.2, indicating a high correlation between experimental values and predicted values, and the response surface design is reasonable. According to the F value and *p*-value, the order of influence on flexibility is: the amount of cellulose ether (C) > the ratio of propylene glycol to glycerol (B) > the mass ratio of vinyl alcohol polymer to microbial polysaccharides (A). The interactions between any two independent variables were not significant (*p* > 0.05).

### 3.3. Validation Experiment

The optimal preparation conditions optimized by this experimental design were as follows: the mass ratio of vinyl alcohol polymer to microbial polysaccharide 17:8 (425 mg:200 mg), the ratio of propylene glycol to glycerol 1:1 (200 mg:200 mg) and the amount of cellulose ether 75 mg. The optimization goal was defined as minimizing disintegration time while targeting flexibility score = 3 (scores < 3 indicate brittleness, while scores > 3 indicate excessive softness, and 4–5 are associated with stickiness). Under these conditions, three replicate experiments were performed. The disintegration time of Jujuboside oral films was 25 s, with a relative deviation of 4.00% from the theoretical predicted value of 24.509 s. The flexibility was 2.57, with a relative deviation of 2.25% from the theoretical predicted value of 2.536. The experimental flexibility (2.57) closely approaches the target optimum of 3, and both relative deviations are below 5%, confirming the reliability of the optimized parameters.

### 3.4. Typical Chromatogram and Retention Time

**Figure 5:**
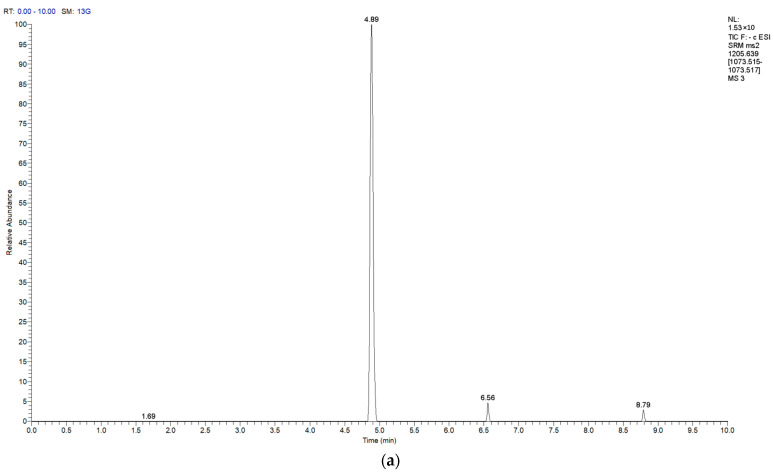

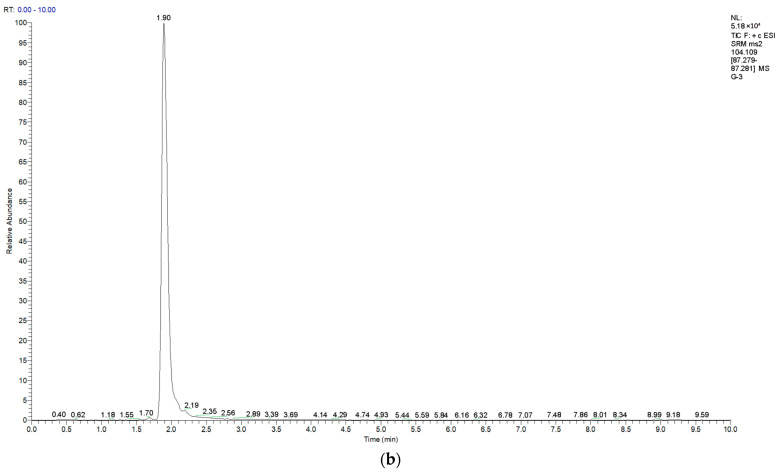
Chromatograms of (**a**) jujuboside A and (**b**) γ-aminobutyric acid.

The MRM chromatograms of jujuboside A and γ-aminobutyric acid are shown in Figure 5. The results in Figure 5a indicate that jujuboside A has a retention time of 4.89 min with stable retention time, symmetrical and sharp peak shape and no interference from impurity peaks. The results in Figure 5b show that γ-aminobutyric acid has a retention time of 1.90 min with stable retention time, symmetrical and sharp peak shape, good separation from adjacent impurity peaks and a flat baseline. The above results demonstrate that this method possesses good specificity and stability.

### 3.5. Confirmation by Primary and Secondary Mass Spectrometry

**Figure 6:**
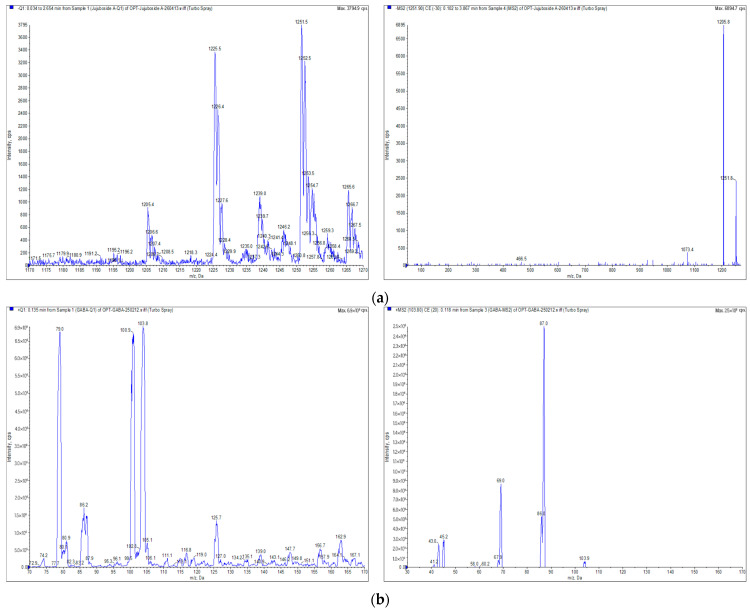
MS^1^ and MS^2^ spectra of (**a**) jujuboside A and (**b**) γ-aminobutyric acid.

The MS^1^ spectrum of jujuboside A (Figure 6a) in negative ion mode showed the most intense ion peak at *m*/*z* 1251.5. In addition, ion peaks at *m*/*z* 1225.5, 1226.4 and 1252.5 were also detected, which may correspond to isotopic peaks or other adduct forms. The MS^2^ spectrum (Figure 6a) was acquired using *m*/*z* 1251.9 as the precursor ion under collision-induced dissociation. The major fragment ion was observed at *m*/*z* 1205.8, corresponding to [M − H]^−^ resulting from neutral loss of formic acid (HCOOH, 46 Da) from the precursor ion. In addition, residual precursor ion at *m*/*z* 1251.8 was also detected. No other characteristic fragments were observed. This fragmentation pattern is consistent with the typical behavior of formate adducts, further confirming the compound as jujuboside A.

The MS^1^ spectrum of γ-aminobutyric acid (Figure 6b) in positive ion mode showed the most intense ion peak at *m*/*z* 103.8, assigned to [M + H]^+^ (theoretical value 104.07). Additionally, low-abundance ion peaks at *m*/*z* 100.9, 79.0 and others were observed, likely arising from in-source fragmentation or impurities. The main peak exhibited a high signal-to-noise ratio and did not interfere with the identification of the target compound. The MS^2^ spectrum (Figure 6b) was acquired using the precursor ion at *m*/*z* 103.8 ([M + H]^+^) under collision-induced dissociation. The major fragment ions were observed at *m*/*z* 87.0 ([M + H-NH_3_]^+^) and *m*/*z* 69.0 ([M + H-NH_3_-H_2_O]^+^), with the highest abundance. In addition, low-mass fragments such as *m*/*z* 86.0, 43.0, and 45.2 were also detected. This fragmentation pattern is in complete agreement with the reported cleavage pattern of γ-aminobutyric acid, confirming the compound as γ-aminobutyric acid.

### 3.6. Targeted Quantitative Analysis Results

Targeted quantitative analysis was performed using multiple reaction monitoring (MRM) mode; the results are shown in Table 6. In five batches of oral films, the content of γ-aminobutyric acid ranged from 8.92 to 10.31 μg/g, with an average content of 9.55 ± 0.580 μg/g (range: 1.39 μg/g); the content of jujuboside A ranged from 0.01 to 0.02 μg/g, with an average content of 0.014 ± 0.0055 μg/g (range: 0.01 μg/g). The results indicated good uniformity of content among different batches.

## 4. Discussion

Insomnia is a serious health problem, and improving sleep quality is of substantial public health importance [23]. Studies have shown that approximately 10–30% of the general population suffers from insomnia [24]. Of the general population, 25.3% were dissatisfied with their sleep quality [25]. As sleep quality deteriorates into insomnia, individuals may seek pharmacological assistance. However, dependence and side effects of these medications have drawn much attention. A large number of natural products from traditional Chinese medicine interventions are increasingly being sought [26]. Jujuboside, a component of a traditional Chinese herbal remedy, has been widely and effectively used for thousands of years in Asia to treat insomnia and anxiety [27]. However, factors such as gastric pH, enzyme activity (e.g., pepsin), and gastric emptying rate can affect drug stability in the stomach and subsequent pharmacokinetics [28]. In addition, the presence of hepatic first-pass metabolism can also lead to reduced drug bioavailability [29]. Therefore, the oral mucosa represents an ideal surface for drug delivery. A wide range of active substances can be effectively absorbed through the oral route, offering an alternative to enteral administration while bypassing the harsh gastrointestinal environment and hepatic first-pass metabolism. Oral mucosal drug delivery offers advantages such as convenience, safety, rapid pharmacological response, and applicability to a broader range of drugs, along with improved adherence in children, elderly individuals, and patients with dysphagia [30]. Compared with liquid formulations (e.g., gargles and sprays), local injections, and semi-solid formulations, oral films provide distinct advantages—including enhanced adhesion, resistance to salivary washout, noninvasiveness, user-friendliness, and more sustained drug retention and release [31,32,33]. As a result, many novel multifunctional oral films have emerged in recent years. These studies provide the theoretical basis for the development of oral films and also serve as the main foundation for this study. Therefore, in this study, the mass ratio of vinyl alcohol polymers to microbial polysaccharides, the ratio of propylene glycol to glycerol, and the amount of cellulose ether were first selected as single factors to investigate their effects on the disintegration time and flexibility of Jujuboside Oral Film. The results showed that the mass ratio of vinyl alcohol polymers to microbial polysaccharides, the ratio of propylene glycol to glycerol, and the amount of cellulose ether had significant effects on the disintegration time and flexibility of Jujuboside Oral Film. Accordingly, based on the single-factor experiments, the response surface methodology was used to design and optimize the preparation method of Jujuboside Oral Film. The mass ratio of vinyl alcohol polymers to microbial polysaccharides (A), the ratio of propylene glycol to glycerol (B), and the amount of cellulose ether (C) were selected as factors influencing the optimization of Jujuboside Oral Film preparation, with the disintegration time and flexibility of Jujuboside Oral Film as the response values. A three-factor, three-level experiment was conducted according to the Central composite design. The response surface analysis results showed that the order of influence on the disintegration time was: the amount of cellulose ether (C) > the mass ratio of vinyl alcohol polymer to microbial polysaccharides (A) > the ratio of propylene glycol to glycerol (B). The interactions between A and B, and between A and C were significant. The order of influence on flexibility was: the amount of cellulose ether (C) > the ratio of propylene glycol to glycerol (B) > the mass ratio of vinyl alcohol polymer to microbial polysaccharides (A). The interactions between any two independent variables were not significant. The optimal preparation conditions optimized by this experimental design were as follows: the mass ratio of vinyl alcohol polymer to microbial polysaccharide 17:8 (425 mg:200 mg), the ratio of propylene glycol to glycerol 1:1 (200 mg:200 mg) and the amount of cellulose ether 75 mg. In the validation experiment, the disintegration time of Jujuboside oral films was 25 s, with a relative deviation of 4.00% from the theoretical predicted value of 24.509 s. The flexibility was 2.57, with a relative deviation of 2.25% from the theoretical predicted value of 2.536. These results indicate that the parameters obtained through response surface optimization are reliable.

To further assess the content uniformity of jujuboside oral films prepared under the optimal conditions, we developed and validated an LC-MS/MS method for quantifying jujuboside A and γ-aminobutyric acid, and then applied it to determine the contents in five batches of film samples. The results showed that the chromatographic peaks of jujuboside A and γ-aminobutyric acid were symmetric with stable retention times (4.89 min for jujuboside A and 1.90 min for γ-aminobutyric acid), and no impurity peak interference was observed, indicating that this method has good specificity. The MS1 and MS2 confirmation results further support the accuracy of the compound structures. In negative ion mode, jujuboside A exhibits the [M + HCOO]^−^ adduct ion (*m*/*z* 1251.5) as the characteristic precursor ion. In its MS/MS spectrum, the predominant fragment ion is [M − H]^−^ (*m*/*z* 1205.8), generated by the loss of formic acid (46 Da). This fragmentation behavior is a typical characteristic of saponins forming formate adduct ions in ESI negative ion mode, which has been widely reported in the mass spectrometric analysis of saponins [34,35]. γ-Aminobutyric acid was detected in positive ion mode as the [M + H]^+^ ion (*m*/*z* 103.8). In the MS/MS spectrum, the major fragment ions were observed at *m*/*z* 87.0 ([M + H-NH_3_]^+^) and *m*/*z* 69.0 ([M + H-NH_3_-H_2_O]^+^), which are entirely consistent with the fragmentation pathways reported in the literature, further confirming the structure of this compound [36]. The quantitative results showed that the average content of γ-aminobutyric acid in the five batches of oral films was 9.55 ± 0.580 μg/g, with low batch-to-batch variation, indicating uniform distribution of this component among different batches. For jujuboside A, the response values of all batches were below the limit of quantitation of the method, precluding accurate quantification. Consequently, the numerical values listed in Table 6 are merely apparent concentrations derived from the calibration curve and do not represent true quantitative results; they should not be used for any statistical evaluation of content uniformity. However, jujuboside A was consistently detected in all batches, and the apparent contents were at very low levels (approximately 0.01–0.02 μg/g) with negligible inter-batch differences, suggesting that the distribution of this component in the formulation was also uniform. The extremely low content of jujuboside A is mainly attributed to the low initial purity (only 4%) of this component in the raw material, which limits the amount of active ingredient contributed per unit mass of the raw material. Nevertheless, the consistently low content across batches indicates that the preparation process of the oral films enabled uniform dispersion of jujuboside A without significant batch-to-batch fluctuations, demonstrating good feasibility and reproducibility of the process. Future studies may increase the absolute content of jujuboside A in the formulation by improving the purity of the raw material, thereby enabling accurate quantification and further pharmacodynamic evaluation. In addition, further in vitro release studies can be carried out (e.g., using the paddle-over-disk method or the dialysis bag method) to simulate release conditions in the oral environment. The cumulative release of jujuboside A and γ-aminobutyric acid from the oral film at different time points should be determined and the release profiles can be fitted using kinetic models. The drug release mechanism of the oral film can then be elucidated through the parameters derived from the release kinetics [37,38].

## 5. Conclusions

The optimal preparation conditions obtained by response surface optimization were mass ratio of vinyl alcohol polymer to microbial polysaccharide of 17:8 (425 mg:200 mg), ratio of propylene glycol to glycerol of 1:1 (200 mg:200 mg) and cellulose ether amount of 75 mg. Under these conditions, three replicate validation experiments were conducted. The disintegration time of the oral films was 25 s, with a relative deviation of 4.00% from the theoretical predicted value, and the flexibility was 2.57, with a relative deviation of 2.25% from the theoretical predicted value. Additionally, the content uniformity of the active ingredients in the films was satisfactory. These results indicate that the process parameters optimized by response surface methodology are reliable and exhibit good repeatability.

## Figures and Tables

**Table 1:** Rating Standard for Film Flexibility.

Film Score	Flexibility Description
1	Insufficient—Extremely brittle; breaks immediately upon any bending (angle < 30°) with no deformation before failure
2	Slightly insufficient—Brittle; breaks when bent to 30–60°, showing minimal plastic deformation before cracking
3	Optimal—Ideal flexibility; can be bent to 180° without any cracks or permanent deformation; exhibits good elastic recovery upon release
4	Slightly excessive—Over-flexible; can be bent beyond 180° but shows noticeable sagging, poor recovery, or tendency to curl under its own weight
5	Excessive—Over-soft and floppy; cannot maintain a flat planar shape; deforms permanently under gravity or slight handling

**Table 2:** Factors and Levels in the Central Composite Design.

Factor	Level				
	−α (Axial Low)	−1 (Low)	0 (Center)	1 (High)	+α (Axial High)
A: The mass ratio of vinyl alcohol polymers to microbial polysaccharides	2.66:2.34	3:2	3.5:1.5	4:1	4.34:0.66
B: The ratio of propylene glycol to glycerol	−0.68:4.68 *	0:4	1:3	1:1	2.68:1.32
C: The amount of cellulose ether (mg)	40.91	75	125	175	209.09

* For factor B, the negative value at the −α level is a mathematical extrapolation in the coded space; in actual preparation, the minimum ratio was set to 0:4 (pure glycerol), corresponding to the −1 level.

**Table 3:** Results of the Response Surface Experiment Design.

Run	Independent Variable			Response Variable	
	A: The Mass Ratio of Vinyl Alcohol Polymers to Microbial Polysaccharides	B: The Ratio of Propylene Glycol to Glycerol	C: The Amount of Cellulose Ether (mg)	Disintegration Time (s)	Flexibility
1	3:2	0:4	175	105	1
2	3.5:1.5	1:3	125	45	1.5
3	3:2	1:1	75	32	2
4	4.34:0.66	1:3	125	72	1.8
5	3.5:1.5	1:3	125	54	1.2
6	4:1	0:4	175	49	1
7	4:1	1:1	175	68	1.5
8	3.5:1.5	1:3	40.9104	14	3.1
9	3.5:1.5	1:3	125	48	1.2
10	3:2	0:4	75	43	1.4
11	3.5:1.5	1:3	125	47	1.5
12	2.66:2.34	1:3	125	86	1
13	4:1	0:4	75	24	2
14	3.5:1.5	−0.68:4.68	125	49	1.5
15	3.5:1.5	2.68:1.32	125	33	2.4
16	4:1	1:1	75	46	2.9
17	3:2	1:1	175	74	1
18	3.5:1.5	1:3	209.09	90	1
19	3.5:1.5	1:3	125	50	1.4
20	3.5:1.5	1:3	125	45	1.2

**Table 4:** Analysis of Variance Table for Disintegration Time of Jujuboside Oral Films.

Source	Sum of Squares	df	Mean Square	F Value	*p*-Value
Model	9467.08	9	1051.90	35.64	<0.0001
A-The mass ratio of vinyl alcohol polymers to microbial polysaccharides	600.32	1	600.32	20.34	0.0011
B-The ratio of propylene glycol to glycerol	57.03	1	57.03	1.93	0.1946
C-The amount of cellulose ether	5692.27	1	5692.27	192.88	<0.0001
AB	861.13	1	861.13	29.18	0.0003
AC	406.13	1	406.13	13.76	0.0040
BC	66.13	1	66.13	2.24	0.1653
A^2^	1534.21	1	1534.21	51.99	<0.0001
B^2^	140.03	1	140.03	4.74	0.0544
C^2^	8.59	1	8.59	0.29	0.6014
Residual	295.12	10	29.51		
Lack of Fit	236.29	5	47.26	4.02	0.0766
Pure Error	58.83	5	11.77		
Cor Total	9762.20	19			

**Table 5:** Analysis of Variance Table for Flexibility of Jujuboside Oral Films.

Source	Sum of Squares	df	Mean Square	F Value	*p*-Value
Model	6.97	9	0.77	21.50	<0.0001
A-The mass ratio of vinyl alcohol polymers to microbial polysaccharides	0.82	1	0.82	22.75	0.0008
B-The ratio of propylene glycol to glycerol	0.90	1	0.90	25.10	0.0005
C-The amount of cellulose ether	3.94	1	3.94	109.27	<0.0001
AB	0.080	1	0.080	2.22	0.1670
AC	0.13	1	0.13	3.47	0.0921
BC	0.13	1	0.13	3.47	0.0921
A^2^	8.727 × 10^−3^	1	8.727 × 10^−3^	0.24	0.6332
B^2^	0.42	1	0.42	11.54	0.0068
C^2^	0.61	1	0.61	16.85	0.0021
Residual	0.36	10	0.036		
Lack of Fit	0.25	5	0.049	2.18	0.2065
Pure Error	0.11	5	0.023		
Cor Total	7.33	19			

**Table 6:** Targeted Quantitative Analysis Results of Jujuboside Oral Films.

Sample	Sample ID	Content (μg/g)	Mean (μg/g)	Variance (μg/g)^2^	SD (μg/g)
Jujuboside A	1	0.01	0.014	3.00 × 10^−5^	0.0055
	2	0.02			
	3	0.02			
	4	0.01			
	5	0.01			
γ-Aminobutyric acid	1	9.32	9.55	0.336	0.580
	2	10.31			
	3	10.00			
	4	8.92			
	5	9.21			

## Data Availability

The original contributions presented in this study are included in the article. Further inquiries can be directed to the corresponding authors.
